# The clinical features and prognostic implications of *PTPN11* mutation in adult patients with acute myeloid leukemia in China

**DOI:** 10.1002/cam4.6669

**Published:** 2023-11-08

**Authors:** Jinjun Yang, Lei Zhao, Yu Wu, Ting Niu, Yuping Gong, Xinchuan Chen, Xiaoou Huang, Jiazhuo Liu, Yang Dai, Hongbing Ma

**Affiliations:** ^1^ Department of Hematology and Institute of Hematology, West China Hospital Sichuan University Chengdu China

**Keywords:** acute myeloid leukemia, event‐free survival, overall survival, *PTPN11*

## Abstract

**Background:**

The clinical significance of protein tyrosine phosphatase nonreceptor type 11 mutation (*PTPN11* ^mut^) in acute myeloid leukemia (AML) is underestimated.

**Methods:**

We collected the data of AML patients with mutated *PTPN11* and wild‐type *PTPN11* (*PTPN11* ^wt^) treated at our hospital and analyzed their clinical characteristics and prognosis.

**Results:**

Fifty‐nine *PTPN11* ^mut^ and 124 *PTPN11* ^wt^ AML patients were included. *PTPN11* ^mut^ was more common in myelomonocytic and monocytic leukemia, and was more likely to co‐mutate with *KRAS*, *KMT2C*, *NRAS*, *U2AF1*, *NOTCH1*, *IKZF1*, and *USH2A* mutations than *PTPN11* ^wt^. The overall survival for AML patients with *PTPN11* ^mut^ was significantly shorter than that for those with *PTPN11* ^wt^ (*p* = 0.03). The negative impact of *PTPN11* ^mut^ on overall survival was pronounced in the “favorable” and “intermediate” groups of ELN2017 risk stratification, as well as in the wild‐type *NPM1* group (*p* = 0.01, *p* = 0.01, and *p* = 0.04).

**Conclusion:**

*PTPN11* ^mut^ is associated with distinct clinical and molecular characteristics, and adverse prognosis in AML patients.

## 1 INTRODUCTION

Acute myeloid leukemia (AML) is a hematologic malignancy caused by clonal proliferation and differentiation arrest of myeloid precursors.^1^ Somatic mutations, such as *NPM1*, *FLT3‐ITD*, and *TP53*, enable hematopoietic stem and progenitor cells to acquire the ability of self‐renewal, which is crucial for the pathogenesis and prognosis of AML.^2^

Mutation in tyrosine‐protein phosphatase nonreceptor type 11 (*PTPN11* ^mut^) can be found in 1.5%–12% of adult AML patients.^3^ The *PTPN11* gene, located on chromosome 12q24, encodes a protein composed of a N‐terminal Src homology 2 (N‐SH2), a protein tyrosine phosphatase (PTP) catalytic domain and a C‐terminal SH2.^4^ N‐SH2 can prevent the PTP domain from being overactivated.^5^ In leukemogenesis, *PTPN11* ^mut^ blocks self‐inhibition of SH2 catalytic activity, which induces increased sensitivity of hematopoietic stem and progenitor cells to granulocyte‐macrophage colony‐stimulating factor and RAS signaling pathway hyperactivation, leading to leukemic transformation.^6^, ^7^, ^8^, ^9^

Currently, the prognosis of *PTPN11* ^mut^ in AML is controversial. Some studies showed that *PTPN11* ^mut^ was associated with an adverse prognosis,^10^, ^11^, ^12^, ^13^ while another report uncovered that AML patients with *PTPN11* ^mut^ presented an improved prognosis.^14^ In addition, other studies did not observe a significant prognostic effect of *PTPN11* ^mut^ in AML.^15^, ^16^ Hence, the prognostic value of *PTPN11* ^mut^ in AML needs further exploration. Herein, we report a single‐center data from China to compare the clinical and molecular features, and outcomes between adult AML patients with mutated *PTPN11* and wild‐type *PTPN11* (*PTPN11* ^wt^).

## 2 METHODS

We retrospectively collected the data of adult AML patients with *PTPN11* ^mut^ (≥18 years of age) from West China Hospital between May 2015 and October 2022. Of 1531 adult AML patients, *PTPN11* mutations were detected in 59 patients (3.9%). Among these 59 *PTPN11* ^mut^ patients, 41 patients received chemotherapy. Then, patients with *PTPN11* ^wt^ were randomly included in a 3:1 ratio to patients with *PTPN11* ^mut^ who received chemotherapy based on diagnosis year, age, sex, and ELN2017 risk stratification. Detailed information about treatment, outcome indicators, detection methods for cytogenetics and molecular biology, and statistical analysis are presented in the Appendix S1 (Figure S4).

## 3 RESULTS

### 3.1 Baseline characteristics

The baseline characteristics of the *PTPN11* ^mut^ and *PTPN11* ^wt^ groups were similar and are shown in Table 1. Patients with *PTPN11* ^mut^ were significantly more common in acute myelomonocytic and monocytic leukemia (AMML/AMOL) than those with *PTPN11* ^wt^ (53.7% vs. 35.5%, *p* = 0.04).

**TABLE 1:** The clinical and molecular characteristics of AML patients with *PTPN11* ^mut^ and *PTPN11* ^wt^.

Characteristic	*PTPN11* ^mut^ (*n* = 41)	*PTPN11* ^wt^ (*n* = 124)	*p* value
Age (years)	54.0 (19.0–75.0)	50.0 (16.0–76.0)	0.52
Sex			
Female	25.0 (61.0)	83.0 (66.9)	0.49
Male	16.0 (39.0)	41.0 (33.1)	
Disease status			
De novo AML	35.0 (85.4)	116.0 (93.5)	0.19
sAML/t‐AML	6.0 (14.6)	8.0 (6.5)	
Laboratory			
WBC (×10^9^/L)	15.4 (0.6–182.4)	17.7 (0.3–286.8)	0.91
ANC (×10^9^/L)	1.0 (0–42.3)	0.9 (0–24.6)	0.58
Platelet (×10^9^/L)	63.0 (8.0–634.0)	48.5 (2.0–429.0)	0.16
Hemoglobin (g/dL)	7.4 (3.9–12.3)	7.8 (3.1–13.0)	0.65
LDH (IU/L)	342.5 (136.0–3466.0)	395.0 (118.0–4410.0)	0.83
PB blasts (%)	25.0 (0–99.0)	29.0 (0–99.0)	0.84
BM blasts (%)	51.5 (7.0–96.0)	57.0 (7.0–94.0)	0.47
AMML/AMOL	22.0 (53.7)	44.0 (35.5)	0.04
Extramedullary involvement	4.0 (9.8)	7.0 (5.6)	0.58
Cytogenetics			
Normal	19.0 (46.3)	62.0 (50.0)	0.69
Complex	7.0 (17.1)	11.0 (8.9)	0.24
t(8;21)(q22;q22)	0 (0)	11.0 (8.9)	0.11
inv(16)(p13q22)/t(16;16)(p13;q22)	3.0 (7.3)	8.0 (6.5)	1.00
inv(3)(q21q26)/t(3;3)(q21;q26)	2.0 (4.9)	0 (0)	0.06
−7	1.0 (2.4)	0 (0)	0.25
+8	0 (0)	5.0 (4.0)	0.44
Others	4.0 (9.8)	19.0 (15.3)	0.37
Insufficient	5.0 (12.2)	8.0 (6.5)	0.40
ELN‐2017 Risk			
Favorable	18.0 (43.9)	47.0 (37.9)	0.71
Intermediate	8.0 (19.5)	31.0 (25.0)	
Adverse	15.0 (36.6)	46.0 (37.1)	
Treatment			
High intensity	29 (70.7)	102 (82.3)	0.11
Low intensity	12 (29.3)	22 (17.7)	
BM transplant			
allo‐HSCT	6.0 (14.6)	22.0 (17.7)	0.65
auto‐HSCT	0 (0)	1.0 (0.8)	1.00
CR	25 (61.0)	84 (67.7)	0.43

*Note*: Values are *n* (%) or median (range).

Abbreviations: allo‐HSCT, allogeneic hematopoietic stem cell transplantation; AML, acute myeloid leukemia; AMML/AMOL, acute myelomonocytic and monocytic leukemia; ANC, absolute neutrophil count; auto‐HSCT, autologous hematopoietic stem cell transplantation; BM, bone marrow; CR, complete remission; ELN, European LeukemiaNet; LDH, lactate dehydrogenase; PB, peripheral blood; *PTPN11* ^mut^, *PTPN11* mutation; *PTPN11* ^wt^, *PTPN11* wild type; sAML, secondary AML; t‐AML, therapy‐related AML; WBC, white blood cell.

### 3.2 Mutation landscape

Seventy‐four *PTPN11* mutations were identified in 59 patients. Forty‐nine (83.1%) patients harbored a single mutation, and 10 (16.9%) carried more than one different *PTPN11* variant (double mutated, *n* = 7; triple mutated, *n* = 1, quadruple mutated, *n* = 2). The multiple mutations among 6 patients were on the same alleles. *PTPN11* mutations were exclusively missense single nucleotide variants. Most mutations (50/74; 67.6%) were localized in the N‐SH2 domain, whereas a minority of mutations (21/74; 28.4%) were localized in the PTP domain. These mutations were localized in exons 3, 8, 12, and 13. A72T, the most common amino acid substitution, was found in eight patients (11.1%) (Figure S1). The median VAF of *PTPN11* ^mut^ patients was 8.6%, ranging from 1.0% to 57.9%.

In addition, patients with *PTPN11* ^mut^ were more likely to co‐mutate with *KRAS* (22.0% vs. 4.8%, *p* < 0.01), *KMT2C* (10.2% vs. 0.8%, *p* < 0.01), *NRAS* (20.3% vs. 8.9%, *p* = 0.03), *U2AF1* (11.9% vs. 4.0%, *p* = 0.045), *NOTCH1* (5.1% vs. 0%, *p* = 0.03), *IKZF1* (5.1% vs. 0%, *p* = 0.03), and *USH2A* (5.1% vs. 0%, *p* = 0.03) mutations than those with *PTPN11* ^wt^ (Figure S2 and Table S1).

### 3.3 Response and survival

**FIGURE 1:**
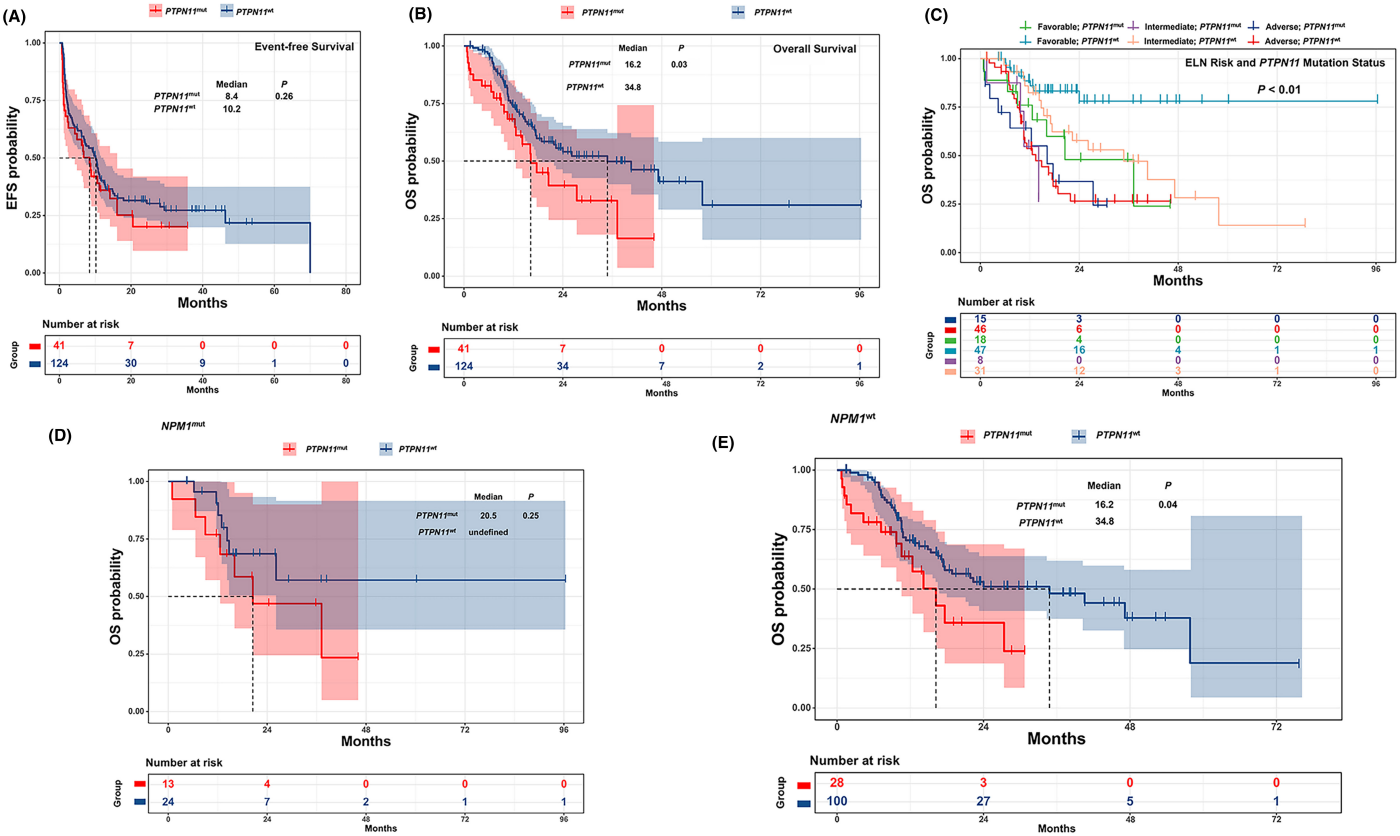
(A) EFS of AML patients with *PTPN11* ^mut^ and *PTPN11* ^wt^. (B) OS of AML patients with *PTPN11* ^mut^ and *PTPN11* ^wt^. (C) OS of AML patients with *PTPN11* ^mut^ and *PTPN11* ^wt^ based on ELN‐2017 risk stratification. (D) OS of *NPM1* ^mut^ AML patients with *PTPN11* ^mut^ and *PTPN11* ^wt^. (E) OS of *NPM1* ^wt^ AML patients with *PTPN11* ^mut^ and *PTPN11* ^wt^.

Although no significant differences were found in complete remission (61.0% vs. 67.7%, *p* = 0.43) or event‐free survival (EFS) (8.4 vs. 10.2 months, *p* = 0.26, Figure 1A) between the *PTPN11* ^mut^ and *PTPN11* ^wt^ groups, patients with *PTPN11* ^mut^ showed shorter overall survival (OS) than patients with *PTPN11* ^wt^ (16.2 vs. 34.8 months, *p* = 0.03, Figure 1B). In addition, the adverse prognosis was pronounced in the “favorable” and “intermediate” groups of ELN2017 (20.5 months vs. undefined, *p* = 0.01; 14.1 vs. 34.8 months, *p* = 0.01; Figure 1C). However, in the “adverse” group, the median OS between patients with *PTPN11* ^mut^ and *PTPN11* ^wt^ was similar (16.2 vs. 13.5 months, *p* = 0.87, Figure 1C).

The impact of the coexistence of *NPM1* mutation (*NPM1* ^mut^) and *PTPN11* ^mut^ on the prognosis of AML patients was also investigated. In the *NPM1* ^mut^ group, there was no difference in OS between patients with *PTPN11* ^mut^ and *PTPN11* ^wt^ (20.5 months vs. undefined, *p* = 0.25, Figure 1D). However, in the *NPM1* wild type (*NPM1* ^wt^) group, patients harboring *PTPN11* ^mut^ had a shorter OS than patients with *PTPN11* ^wt^ (16.2 vs. 34.8 months, *p* = 0.04, Figure 1E).

Moreover, we further studied the prognosis of AMML/AMOL and non‐AMML/AMOL patients with *PTPN11* ^mut^ and *PTPN11* ^wt^. There were no significant differences between patients with *PTPN11* ^mut^ and *PTPN11* ^wt^ in either the AMML/AMOL or non‐AMML/AMOL groups (16.2 vs. 17.4 months, *p* = 0.36; 20.5 vs. 47.1 months, *p* = 0.07) (Figure S3).

## 4 DISCUSSION

Herein, we report the clinical characteristics, gene mutations, and prognosis of adult AML patients with *PTPN11* ^mut^ and *PTPN11* ^wt^ in a large cohort. We identified that *PTPN11* ^mut^ was independently associated with distinct clinical and molecular features and adverse outcomes in AML patients.

Alfayez et al. reported that *PTPN11* ^mut^ was more commonly associated with the AMML/AMOL subtype.^11^ However, the impacts of *PTPN11* ^mut^ on prognosis in AMML/AMOL and non‐AMML/AMOL patients have not been reported. In our study, patients with *PTPN11* ^mut^ seemed to have a poorer prognosis than patients with *PTPN11* ^wt^ in the non‐AMML/AMOL group (*p* = 0.07). However, in the AMML/AMOL group, both the *PTPN11* ^mut^ and *PTPN11* ^wt^ groups showed similar OS.

Gene mutations are critical for the prognosis of AML patients. Previous studies have shown differences in gene mutation profiles among patients with *PTPN11* ^mut^. In our study, there were seven patients (12%) with *FLT3‐ITD* mutations. Similarly, some studies revealed that the incidence rates of *FLT3‐ITD* in *PTPN11* ^mut^ AML patients were 16%–27%.^11^, ^16^, ^17^ However, Hou et al. identified that *PTPN11* ^mut^ and *FLT3‐ITD* mutations were mutually exclusive. This difference might be related to the smaller sample size of Hou et al.^15^ Moreover, the incidence rates of *NPM1* and *DNMT3A* in AML patients with *PTPN11* ^mut^ are controversial. Some researchers reported high incidence rates of *NPM1* and *DNMT3A* with 60.5%–63% and 37%–56.1% in AML patients with *PTPN11* ^mut^, respectively.^14^, ^16^, ^17^ However, others uncovered that the incidence rates of *NPM1* and *DNMT3A* in AML patients with *PTPN11* ^mut^ were 22%–29% and 24%–27%, respectively.^11^, ^12^ Our study supported the results of the latter. Although the most common mutations coexisting with *PTPN11* ^mut^ in our study were *DNMT3A* and *NPM1*, the incidence rates of these two mutations were only 25% and 25%, respectively. In addition, our study found that patients with *PTPN11* ^mut^ were more likely to harbor *KRAS*, *KMT2C, NRAS*, *U2AF1*, *NOTCH1*, *IKZF1*, and *USH2A* mutations than those with *PTPN11* ^wt^. Given that *PTPN11* can modulate the RAS/MAPK signaling axis,^18^ further in vitro and clinical studies are needed to verify whether *PTPN11* ^mut^ combined with these mutations has a synergistic effect on the occurrence and development of AML.

A few published studies have focused on the relationships between *PTPN11* ^mut^ and the clinical outcomes of AML patients. The impacts of *PTPN11* ^mut^ on OS in AML patients are controversial. Some studies showed that *PTPN11* ^mut^ were associated with shorter OS.^10^, ^11^, ^17^, ^19^ However, others reported that the median OS was similar in both the *PTPN11* ^mut^ and *PTPN11* ^wt^ groups.^15^, ^20^ Metzeler et al.^14^ found that patients with *PTPN11* ^mut^ had relatively favorable survival outcomes. Our study also showed shorter OS in patients with *PTPN11* ^mut^ than in those with *PTPN11* ^wt^. In addition, Alfayez et al. reported that *PTPN11* ^mut^ negatively impacted OS across all ELN risk categories.^11^ However, Stasik et al. reported that the negative impact of *PTPN11* ^mut^ was mainly limited to the ELN favorable risk group.^17^ Our study showed that *PTPN11* ^mut^ was poor prognostic factor for the “favorable” and “intermediate” groups of ELN2017, which can facilitate further stratification of the two groups. Moreover, our subgroup analysis supported the idea that *PTPN11* ^mut^ suggested significantly short OS in AML patients with *NPM1* ^wt^, instead of in patients with *NPM1* ^mut^.^12^, ^15^, ^16^, ^21^ Furthermore, our study showed that no significant difference between *PTPN11* ^mut^ and *PTPN11* ^wt^ patients was observed for EFS, which was similar to the report of Stasik et al.^17^ Prospective trials with larger sample sizes and longer follow‐up periods are warranted to explore the impacts of *PTPN11* ^mut^ on OS and EFS in AML patients.

Our study had some limitations. First, this was a retrospective study, and AML patients with *PTPN11* ^mut^ and *PTPN11* ^wt^ at a 1:3 ratio were included, which may cause selection bias. To reduce the risk of bias, patients were randomly included based on the year of diagnosis, age, sex, and ELN2017 risk stratification in this study. Moreover, our study did not explore the detailed mechanism of *PTPN11* ^mut^ in poor prognosis of AML. Previous studies indicated that the poor prognosis of *PTPN11* ^mut^ may be related to its increased resistance to venetoclax and azacitidine.^22^, ^23^ More prospective clinical and basic studies are required to elucidate the impact of *PTPN11* ^mut^ in AML patients and possible molecular mechanisms in AML progression.

## 5 CONCLUSIONS

This study demonstrates that AML patients with *PTPN11* ^mut^ are associated with distinct clinical characteristics and poor prognosis.

## AUTHOR CONTRIBUTIONS

**Jinjun Yang:** Conceptualization (equal); data curation (equal); formal analysis (equal); investigation (equal); methodology (equal); software (equal); visualization (equal); writing – original draft (equal). **Lei Zhao:** Conceptualization (equal); data curation (equal); investigation (equal); software (equal); visualization (equal). **Yu Wu:** Resources (equal). **Ting Niu:** Resources (equal). **Yuping Gong:** Resources (equal). **Xinchuan Chen:** Resources (equal). **Xiaoou Huang:** Resources (equal). **Jiazhuo Liu:** Resources (equal). **Yang Dai:** Resources (equal). **Hongbing Ma:** Conceptualization (equal); resources (equal); supervision (equal); writing – review and editing (equal).

## FUNDING INFORMATION

The study was funded by 1·3·5 project for disciplines of excellence–Clinical Research Incubation Project, West China Hospital, Sichuan University (No. 2022HXFH040) to Hongbing Ma.

## CONFLICT OF INTEREST STATEMENT

The authors declare no conflicts of interest.

## ETHICS STATEMENT

The study was approved by the West China Hospital Institutional Review Board and in accordance with the Helsinki declaration.

## Supporting information


Figure S1

Figure S2

Figure S3

Figure S4

Table S1

Appendix S1

## Data Availability

All the data of our study can be obtained from the corresponding author.

## References

[1] Newell LF , Cook RJ . Advances in acute myeloid leukemia. BMJ. 2021;375:n2026. doi:10.1136/bmj.n2026 34615640

[2] Papaemmanuil E , Gerstung M , Bullinger L , et al. Genomic classification and prognosis in acute myeloid leukemia. N Engl J Med. 2016;374(23):2209‐2221. doi:10.1056/NEJMoa1516192 27276561 PMC4979995

[3] Tartaglia M , Niemeyer CM , Fragale A , et al. Somatic mutations in PTPN11 in juvenile myelomonocytic leukemia, myelodysplastic syndromes and acute myeloid leukemia. Nat Genet. 2003;34(2):148‐150. doi:10.1038/ng1156 12717436

[4] Pandey R , Saxena M , Kapur R . Role of SHP2 in hematopoiesis and leukemogenesis. Curr Opin Hematol. 2017;24(4):307‐313. doi:10.1097/moh.0000000000000345 28306669 PMC5709049

[5] Hof P , Pluskey S , Dhe‐Paganon S , Eck MJ , Shoelson SE . Crystal structure of the tyrosine phosphatase SHP‐2. Cell. 1998;92(4):441‐450. doi:10.1016/s0092-8674(00)80938-1 9491886

[6] Chan RJ , Leedy MB , Munugalavadla V , et al. Human somatic PTPN11 mutations induce hematopoietic‐cell hypersensitivity to granulocyte‐macrophage colony‐stimulating factor. Blood. 2005;105(9):3737‐3742. doi:10.1182/blood-2004-10-4002 15644411 PMC1895012

[7] Schubbert S , Lieuw K , Rowe SL , et al. Functional analysis of leukemia associated PTPN11 mutations in primary hematopoietic cells. Blood. 2005;106(1):311‐317. doi:10.1182/blood-2004-11-4207 15761018 PMC1895116

[8] Mohi MG , Williams IR , Dearolf CR , et al. Prognostic, therapeutic, and mechanistic implications of a mouse model of leukemia evoked by Shp2 (PTPN11) mutations. Cancer Cell. 2005;7(2):179‐191. doi:10.1016/j.ccr.2005.01.010 15710330

[9] Dong L , Yu WM , Zheng H , et al. Leukaemogenic effects of Ptpn11 activating mutations in the stem cell microenvironment. Nature. 2016;539(7628):304‐308. doi:10.1038/nature20131 27783593 PMC5317374

[10] Kaner JD , Mencia‐Trinchant N , Schaap A , et al. Acute myeloid leukemia (AML) with somatic mutations in PTPN11 is associated with treatment resistance and poor overall survival. Blood. 2018;132:2760.

[11] Alfayez M , Issa GC , Patel KP , et al. The clinical impact of PTPN11 mutations in adults with acute myeloid leukemia. Leukemia. 2021;35(3):691‐700. doi:10.1038/s41375-020-0920-z 32561839

[12] Swoboda DM , Ali NA , Chan O , et al. PTPN11 mutations are associated with poor outcomes across myeloid malignancies. Leukemia. 2021;35(1):286‐288. doi:10.1038/s41375-020-01083-3 33132383

[13] Eisfeld AK , Kohlschmidt J , Mims A , et al. Additional gene mutations may refine the 2017 European LeukemiaNet classification in adult patients with de novo acute myeloid leukemia aged <60 years. Leukemia. 2020;34(12):3215‐3227. doi:10.1038/s41375-020-0872-3 32461631 PMC7882079

[14] Metzeler KH , Rothenberg‐Thurley M , Grlich D , Sauerland MC , Herold T . PTPN11 mutations and outcomes in adult patients with acute myeloid leukemia. Blood. 2020;136(Supplement 1):4‐5.32614961

[15] Hou HA , Chou WC , Lin LI , et al. Characterization of acute myeloid leukemia with PTPN11 mutation: the mutation is closely associated with NPM1 mutation but inversely related to FLT3/ITD. Leukemia. 2008;22(5):1075‐1078. doi:10.1038/sj.leu.2405005 17972951

[16] Fobare S , Kohlschmidt J , Ozer HG , et al. Molecular, clinical, and prognostic implications of PTPN11 mutations in acute myeloid leukemia. Blood Adv. 2022;6(5):1371‐1380. doi:10.1182/bloodadvances.2021006242 34847232 PMC8905707

[17] Stasik S , Eckardt JN , Kramer M , et al. Impact of PTPN11 mutations on clinical outcome analyzed in 1529 patients with acute myeloid leukemia. Blood Adv. 2021;5(17):3279‐3289. doi:10.1182/bloodadvances.2021004631 34459887 PMC8525221

[18] Matozaki T , Murata Y , Saito Y , Okazawa H , Ohnishi H . Protein tyrosine phosphatase SHP‐2: a proto‐oncogene product that promotes Ras activation. Cancer Sci. 2009;100(10):1786‐1793. doi:10.1111/j.1349-7006.2009.01257.x 19622105 PMC11158110

[19] Sun Y , Zhang F , Huo L , et al. Clinical characteristics and prognostic analysis of acute myeloid leukemia patients with PTPN11 mutations. Hematology. 2022;27(1):1184‐1190. doi:10.1080/16078454.2022.2140274 36318614

[20] Loh ML , Reynolds MG , Vattikuti S , et al. PTPN11 mutations in pediatric patients with acute myeloid leukemia: results from the Children's Cancer Group. Leukemia. 2004;18(11):1831‐1834. doi:10.1038/sj.leu.2403492 15385933

[21] Liu J , Qin W , Wang B , et al. PTPN11 mutations in adult acute myeloid leukaemia: prevalence and clinical implications in the context of NPM1 mutation. Leuk Res. 2022;118:106859. doi:10.1016/j.leukres.2022.106859 35617714

[22] Zhang H , Wilmot B , Bottomly D , et al. Biomarkers predicting venetoclax sensitivity and strategies for venetoclax combination treatment. Blood. 2018;132(Supplement 1):175. doi:10.1182/blood-2018-175

[23] Stevens BM , Jones CL , Winters A , et al. PTPN11 mutations confer unique metabolic properties and increase resistance to venetoclax and azacitidine in acute myelogenous leukemia. Blood. 2018;132(Supplement 1):909. doi:10.1182/blood-2018-99-119806

