# Supplementary figures and images for: The clinical features and prognostic implications of PTPN11 mutation in adult patients with acute myeloid leukemia in China
Source: Cancer Med. 2023 Nov 8;12(23):21111–7. doi: 10.1002/cam4.6669 (PMC10726903; doi:10.1002/cam4.6669)

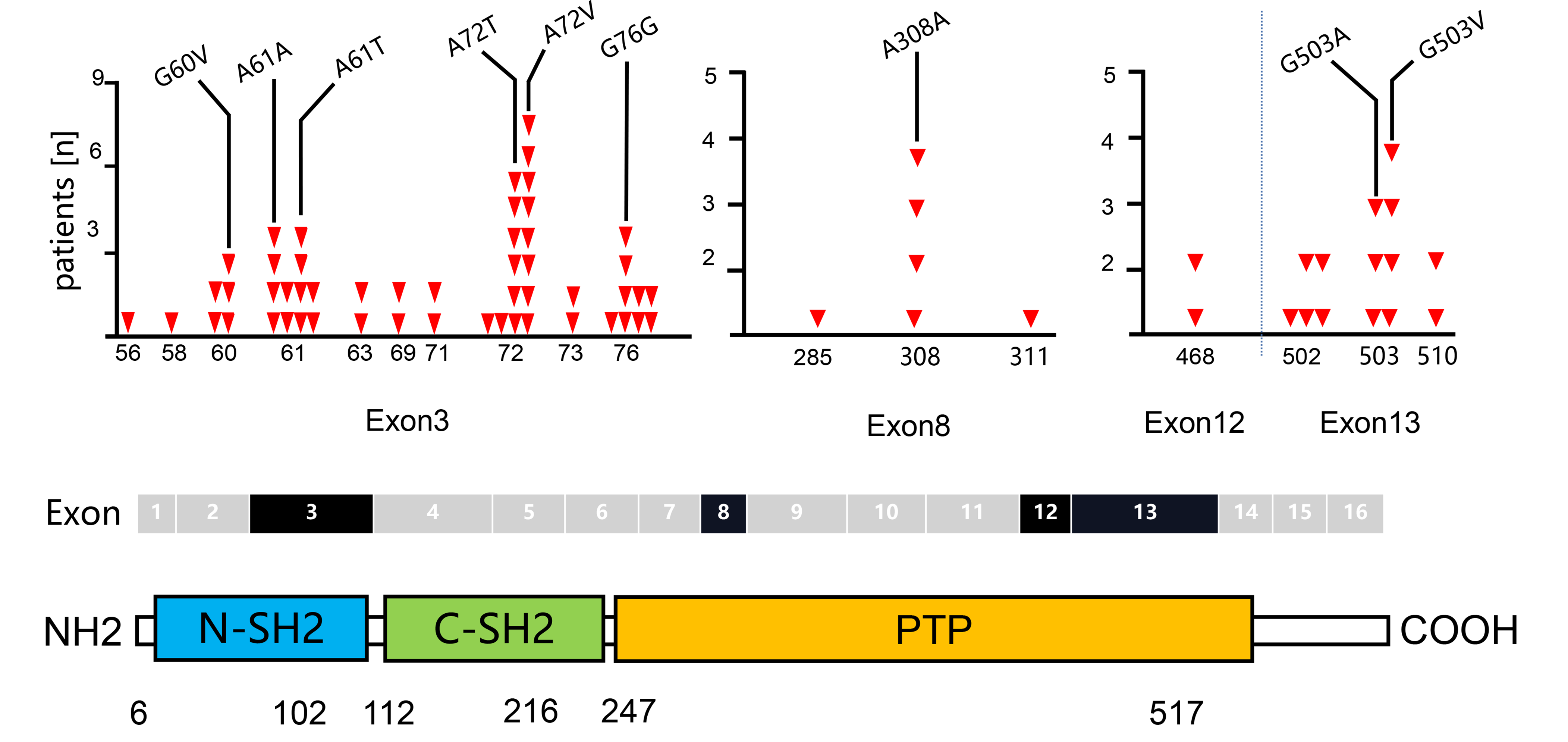

Supplement: Supplementary file 1 — Figure S1 [file CAM4-12-21111-s003.tif]

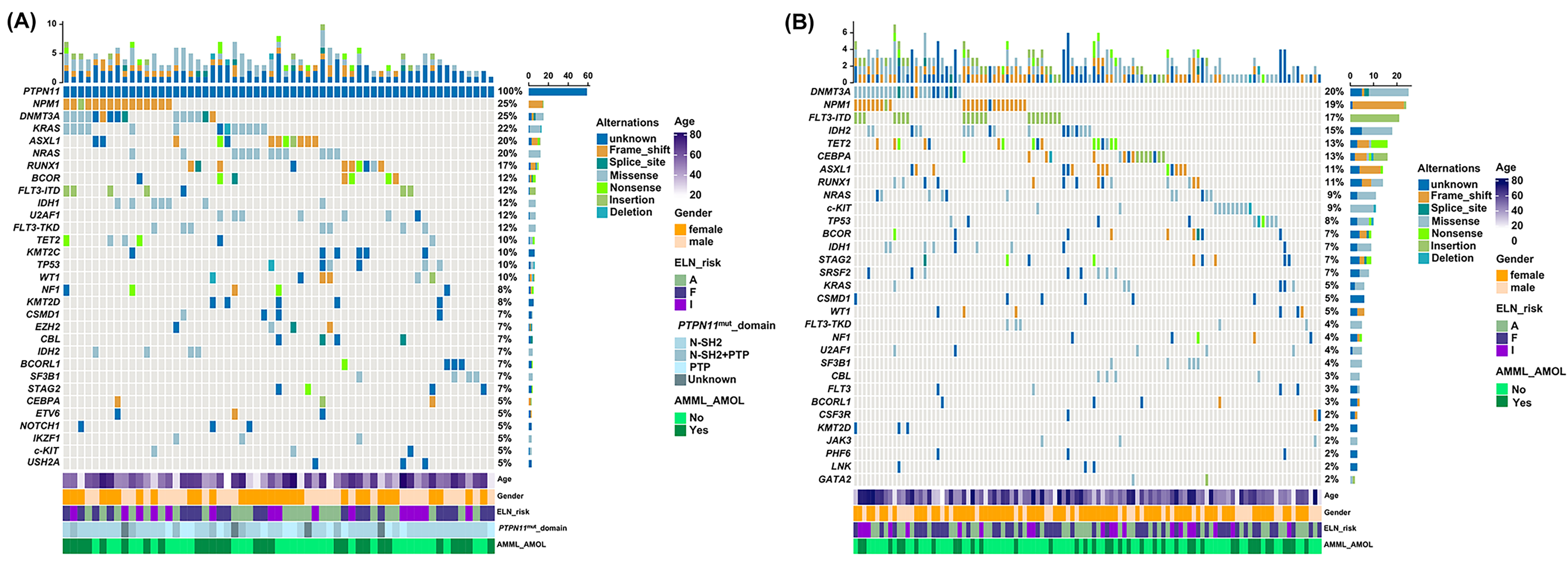

Supplement: Supplementary file 2 — Figure S2 [file CAM4-12-21111-s002.tif]

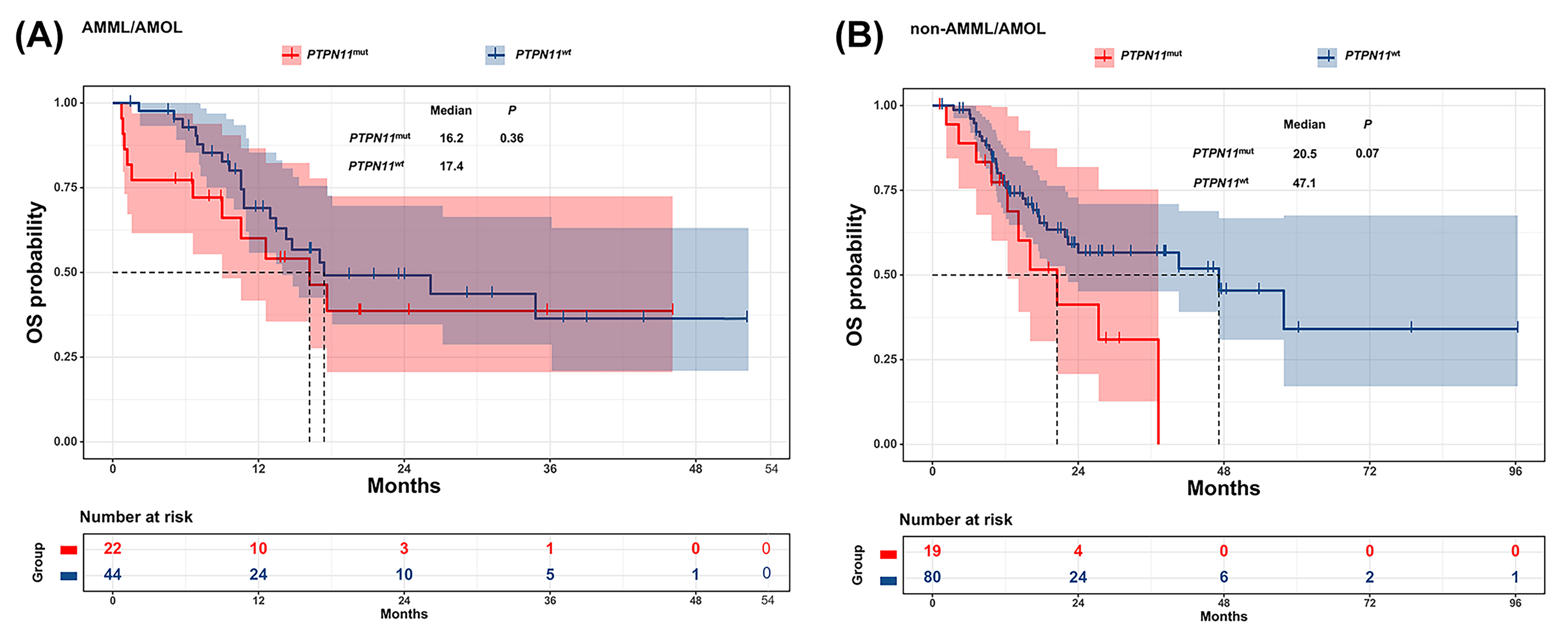

Supplement: Supplementary file 3 — Figure S3 [file CAM4-12-21111-s001.tif]

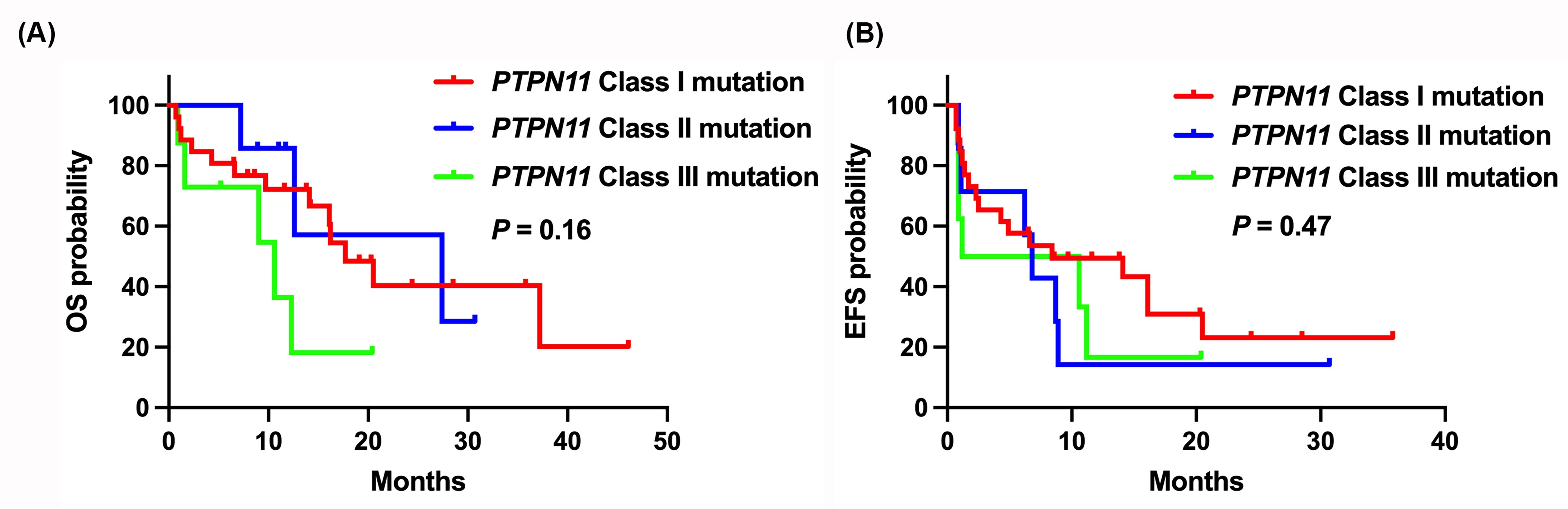

Supplement: Supplementary file 4 — Figure S4 [file CAM4-12-21111-s004.tif]
