# Supplementary material for: The clinical features and prognostic implications of PTPN11 mutation in adult patients with acute myeloid leukemia in China
Source: Cancer Med. 2023 Nov 8;12(23):21111–7. doi: 10.1002/cam4.6669 (PMC10726903; doi:10.1002/cam4.6669)
Supplement: Supplementary file 5 — Table S1 [file CAM4-12-21111-s005.docx]

Table S1. Frequencies of gene mutations in AML patient with *PTPN11*^mut^ and *PTPN11*^wt^

| Gene | *PTPN11*^mut^ (n = 59) | *PTPN11*^wt^ (n = 124) | *P* value |
| --- | --- | --- | --- |
| *NPM1* | 25.4 | 19.4 | 0.35 |
| *DNMT3A* | 25.4 | 20.2 | 0.42 |
| *KRAS* | 22.0 | 4.8 | < 0.01 |
| *ASXL1* | 20.3 | 11.3 | 0.10 |
| *NRAS* | 20.3 | 8.9 | 0.03 |
| *RUNX1* | 16.9 | 11.3 | 0.29 |
| *BCOR* | 11.9 | 6.5 | 0.34 |
| *FLT3-ITD* | 11.9 | 16.9 | 0.37 |
| *IDH1* | 11.9 | 7.3 | 0.30 |
| *U2AF1* | 11.9 | 4.0 | 0.045 |
| *FLT3-TKD* | 11.9 | 4.0 | 0.09 |
| *TET2* | 10.2 | 12.9 | 0.60 |
| *KMT2C* | 10.2 | 0.8 | < 0.01 |
| *TP53* | 10.2 | 8.1 | 0.64 |
| *WT1* | 10.2 | 4.8 | 0.30 |
| *NF1* | 8.5 | 4.0 | 0.38 |
| *KMT2D* | 8.5 | 2.4 | 0.14 |
| *CSMD1* | 6.8 | 4.8 | 0.85 |
| *EZH2* | 6.8 | 0.8 | 0.07 |
| *CBL* | 6.8 | 3.2 | 0.48 |
| *IDH2* | 6.8 | 14.5 | 0.13 |
| *BCORL1* | 6.8 | 3.2 | 0.48 |
| *SF3B1* | 6.8 | 4.0 | 0.66 |
| *STAG2* | 6.8 | 7.3 | 1.00 |
| *CEBPA* | 5.1 | 12.9 | 0.11 |
| *ETV6* | 5.1 | 0.8 | 0.19 |
| *NOTCH1* | 5.1 | 0 | 0.03 |
| *IKZF1* | 5.1 | 0 | 0.03 |
| *c-KIT* | 5.1 | 8.9 | 0.55 |
| *USH2A* | 5.1 | 0 | 0.03 |
| *SRSF2* | 1.7 | 6.5 | 0.31 |
| *CSF3R* | 1.7 | 2.4 | 1.00 |
| *JAK3* | 0 | 2.4 | 0.55 |
| *PHF6* | 3.4 | 2.4 | 1.00 |
| *LNK* | 3.4 | 2.4 | 1.00 |
| *GATA2* | 1.7 | 1.6 | 1.00 |

Abbreviations: Values are %. *PTPN11*^mut^, *PTPN11* mutations; *PTPN11*^wt^, *PTPN11* wild type; AML, acute myeloid leukemia.

**FIGURE LEGENDS**

**FIGURE S1** Schematic showing the locations of *PTPN11*^mut^ in AML patients and the domain structure of *PTPN11*: 2 Src homology 2 domains (N-SH2 and C-SH2) and a protein tyrosine phosphatase domain (PTP). Recurrent alterations and their frequencies in AML patients are indicated for *PTPN11* exons 3, 8, 12, and 13.

**FIGURE S2** Gene mutations in AML patients with *PTPN11*^mut^ (A) and *PTPN11*^wt^ (B). Each column represents an individual patient. Each subsequent row represents a gene. Colored squares indicate the presence of a mutation, and different colors represent different mutation types; gray squares represent no alteration detected. The top row represents the number of mutations in each AML patient.

**FIGURE S3** OS of AML patients with *PTPN11*^mut^ and *PTPN11*^wt^ based on the AMML/AMOL status. (A). the AMML/AMOL patients. (B). the non-AMML/AMOL patients.

**FIGURE S4** OS and EFS of AML patients with *PTPN11* Class I, II, and III mutations. (A). OS. (B) EFS.
