# Supplementary material for: The clinical features and prognostic implications of PTPN11 mutation in adult patients with acute myeloid leukemia in China
Source: Cancer Med. 2023 Nov 8;12(23):21111–7. doi: 10.1002/cam4.6669 (PMC10726903; doi:10.1002/cam4.6669)
Supplement: Supplementary file 6 — Appendix S1 [file CAM4-12-21111-s006.docx]

**Supplementary Materials**

**METHODS**

**Patients and treatment**

The patients were treated with high or low intensity chemotherapy regimens based on the Chinese guidelines for the diagnosis and treatment of adult acute myeloid leukemia (AML).^1-3^ High intensity chemotherapy remains a combination of anthracyclines (usually daunorubicin or idarubicin) and cytosine arabinoside. Low intensity chemotherapy includes low dose cytarabine, hypomethylating agents (decitabine and azacitidine), and other targeted therapies (venetoclax, *FLT3* inhibitors, and *IDH* inhibitors). All patients provided written informed consent for peripheral blood (PB) or bone marrow (BM) sample collection. The study was in accordance with the Helsinki declaration and approved by the West China Hospital Institutional Review Board.

**Cytogenetics and molecular analyses**

Cytogenetics and molecular analyses were performed in the institutional laboratory cooperating with West China Hospital using PB and/or BM samples. Chromosome karyotypes were determined using the G-banding method and analyzed in at least 20 BM metaphase cells. Eighteen common fusion genes in AML were detected by multiplex reverse transcription PCR (RT-PCR) amplification, which was performed as 8 parallel multiplex reactions on a 9700 PCR - Thermal Cycler (Applied Biosystems). *PTPN11* mutation (*PTPN11*^mut^) and other gene mutations of AML were detected with next-generation sequencing (NGS). Hg38 was used as the reference genome to align the reads, and somatic mutations were established by comparing with sequences from the patient's hair follicle and nail to exclude germline mutations. Genomic DNA was extracted and purified using the DNeasy Blood and Tissue Kit (Qiagen, Hilden, Germany), and quantified with the NanoDrop spectrophotometer (Thermo Scientific, Waltham, USA) from fresh PB or BM samples. NGS was performed on clinical-grade, Clinical Laboratory Improvement Amendments-compliant platforms using an Illumina MiSeq system (Illumina, San Diego, USA). The NGS library was prepared using at least 200ng of genomic DNA. The panel targeted 248 genes known to be mutated in myeloid malignancies. A minimum read depth of 2000× (bidirectional true paired-end sequencing) was required. Variant allele frequencies (VAFs) were defined as the ratio of mutant to total reads, and a VAF ≥ 1% was evaluated as positive. For *PTPN11*^mut^, the panel covers all relevant mutational hotspots of the N-SH2, C-terminal SH2 and PTP domain. *FTL3-ITD* was detected using RT-PCR followed by capillary electrophoresis DNA fragment analysis on Applied Biosystems 3500xL Genetic Analyzer. Gene mutations of patients are classified into four categories based on the clinical significance level and grading criteria: I, strong clinical significance; II, potential clinical significance; III, clinical significance unknown; IV, benign and potentially benign.^4^ Sources of gene mutation annotation database include but not limited to: 1000 Genomes Project, ExAc (the Exome Aggregation Consortium), dbSNP (the Single Nucleotide Polymorphism Database), ClinVar (National Center for Biotechnology Information Clinical Disease Related Variants Database), HGMD (Human Gene Mutation Database), Ensenbl (Genome database), UCSC (the genome database established by the University of California, Santa Cruz, USA), and Cosmic (catalogue of somatic mutations in cancer). There were no significant differences on OS and EFS among *PTPN11* with Class I, II, and III mutations (*P* = 0.16, *P* = 0.47; respectively) (Figure S4).

**Statistical analysis**

Patient characteristics were described using medians and ranges for continuous variables, and frequencies and percentages for categorical variables. Continuous variables were compared using t test or nonparametric Mann-Whitney U test. Categorical variables were compared using chi-square tests or Fisher exact tests. CR required no PB blasts cells, less than 5% BM blast cells without Auer rods, and no extramedullary involvement. Overall survival (OS) was defined as the time from diagnosis to death or last follow-up. Event-free survival (EFS) was calculated from diagnosis to first failure, including death, relapse, or treatment abandonment due to disease progression. To evaluate OS and EFS, the Kaplan-Meier method and log-rank test were performed. *P* < 0.05 was considered significant. All statistical analyses were performed with the R environment for statistical computing version 4.2.1.

**REFERENCES**

1. Jishi Wang. Expert Consensus on the Treatment of Acute Myeloid Leukemia 2010:

2. [Chinese guidelines for diagnosis and treatment of adult acute myeloid leukemia (not APL) (2017)]. *Zhonghua Xue Ye Xue Za Zhi*. Mar 14 2017;38(3):177-182. doi:10.3760/cma.j.issn.0253-2727.2017.03.001

3. [Chinese guidelines for the diagnosis and treatment of adult acute myeloid leukemia (not APL) (2021)]. *Zhonghua Xue Ye Xue Za Zhi*. Aug 14 2021;42(8):617-623. doi:10.3760/cma.j.issn.0253-2727.2021.08.001

4. Li MM, Datto M, Duncavage EJ, et al. Standards and Guidelines for the Interpretation and Reporting of Sequence Variants in Cancer: A Joint Consensus Recommendation of the Association for Molecular Pathology, American Society of Clinical Oncology, and College of American Pathologists. *J Mol Diagn*. Jan 2017;19(1):4-23. doi:10.1016/j.jmoldx.2016.10.002
